# Supplementary material for: Ascorbate–Glutathione Oxidant Scavengers, Metabolome Analysis and Adaptation Mechanisms of Ion Exclusion in Sorghum under Salt Stress
Source: Int J Mol Sci. 2021 Dec 9;22(24):13249. doi: 10.3390/ijms222413249 (PMC8704531; doi:10.3390/ijms222413249)
Supplement: Supplementary file 1 [file ijms-22-13249-s001.zip › ijms-1459028-supplementary.pdf]

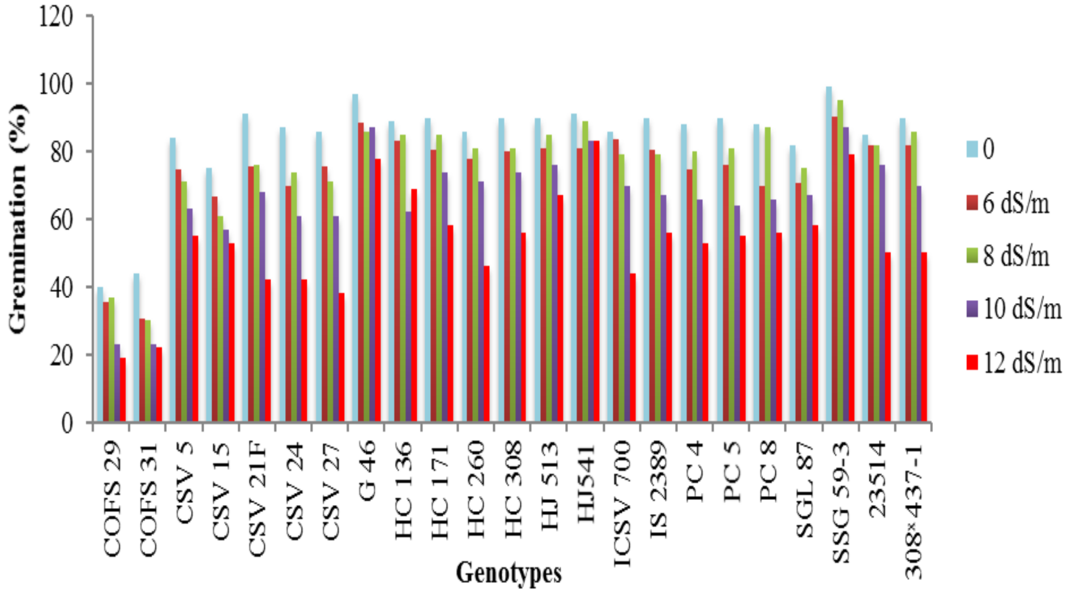

Figure S1. Effect of salt stress on germination (%) in sorghum genotypes

C.D. 1.887 **SE(d)** 0.958 **SE(m)** 1.547

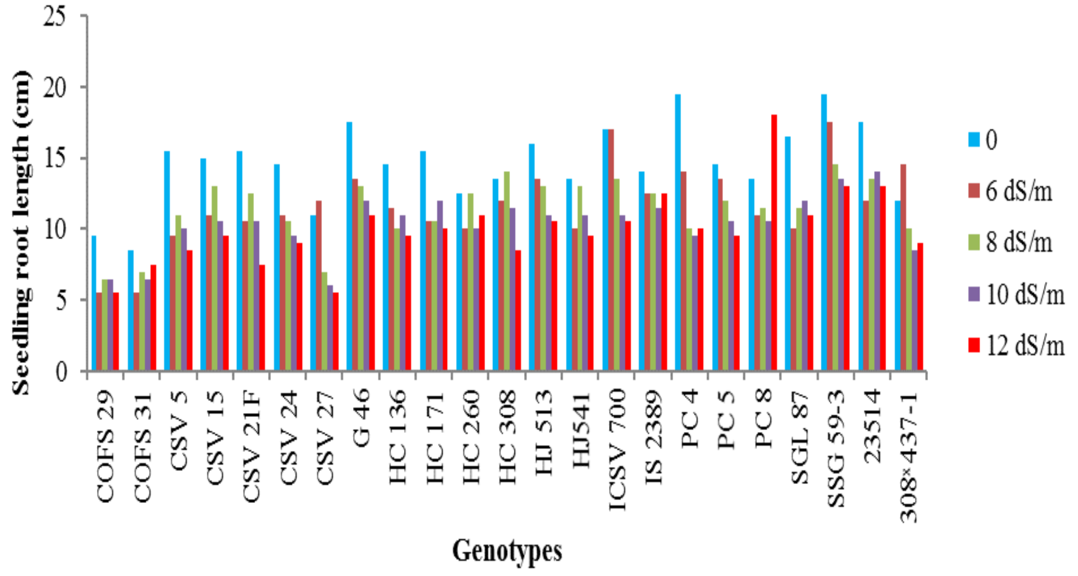

Figure S2. Effect of salt stress on seedling root length (cm) in sorghum genotypes

C.D. 0.689 **SE(d)** 0.350 **SE(m)** 0.247

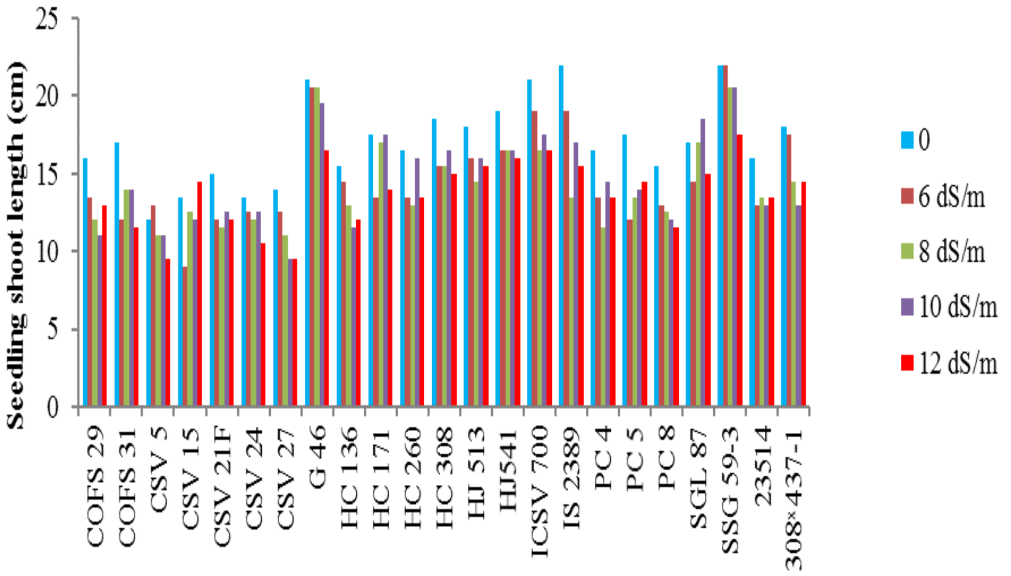

Figure S3. Effect of salt stress on seedling shoot length (cm) in sorghum genotypes

C.D. 0.646 **SE(d)** 0.328 **SE(m)** 0.232

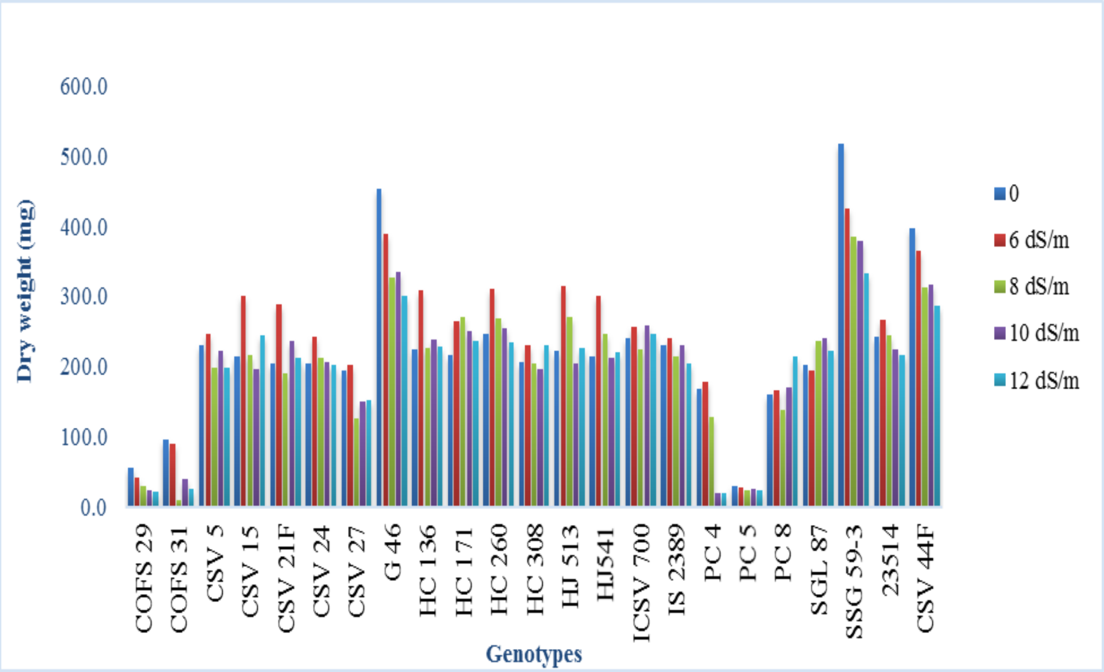

Figure S4. Effect of salt stress on dry seedling weight (mg) in sorghum genotypes

C.D. 1.546 **SE(d)** 0.623 **SE(m)** 0.546

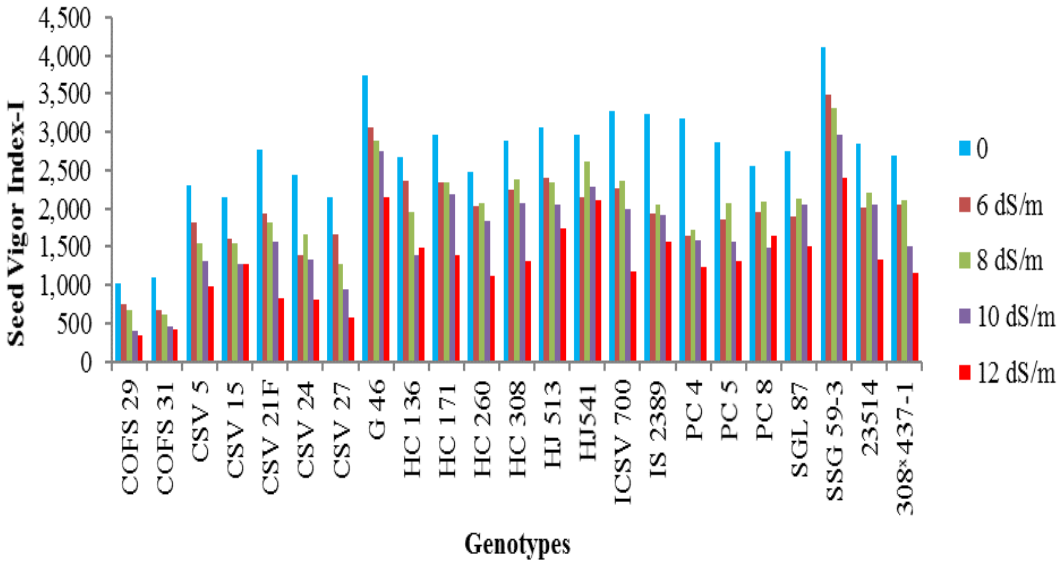

Figure S5. Effect of salt stress on vigor index-I in sorghum genotypes

C.D. 81.311 **SE(d)** 41.280 **SE(m)** 29.190

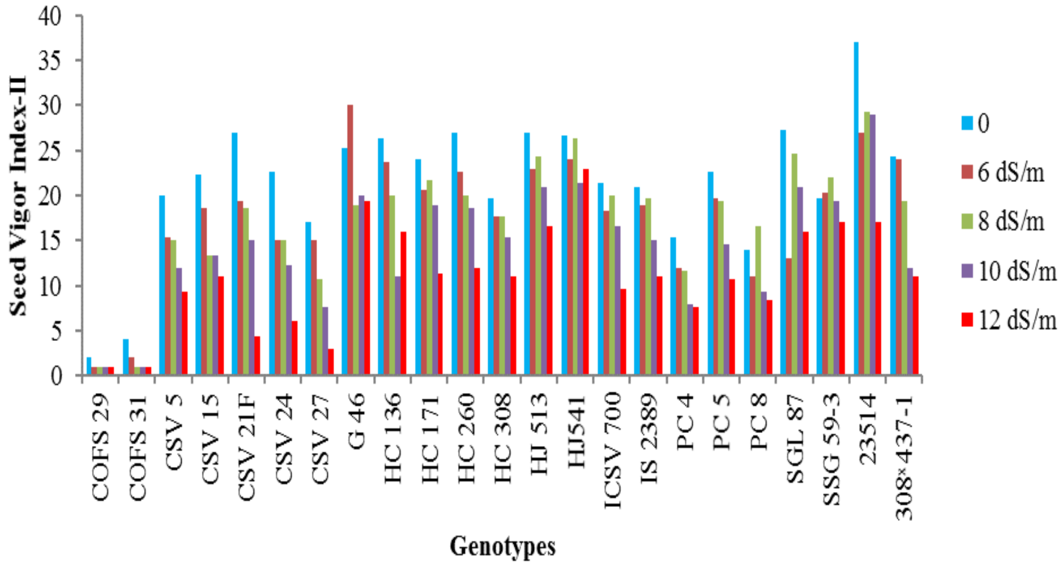

Figure S6. Effect of salt stress on vigor index-II in sorghum genotypes

C.D. 0.838 **SE(d)** 0.426 **SE(m)** 0.301

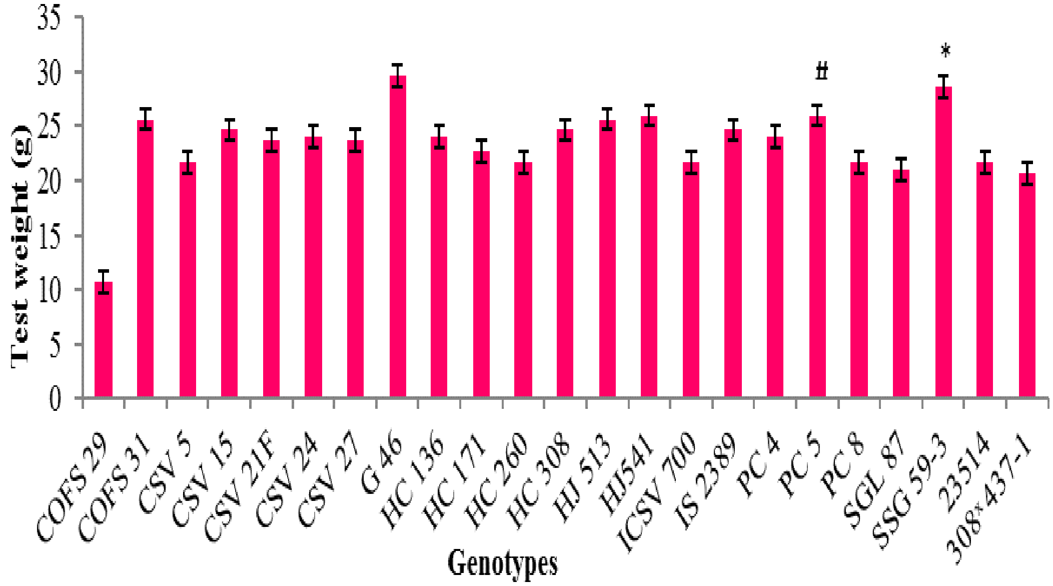

Figure S7. Test weight (g) in sorghum genotypes

C.D. 1.808

(a)

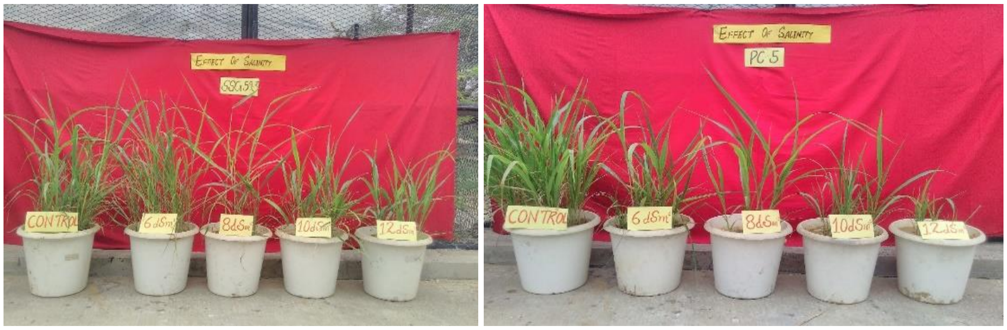

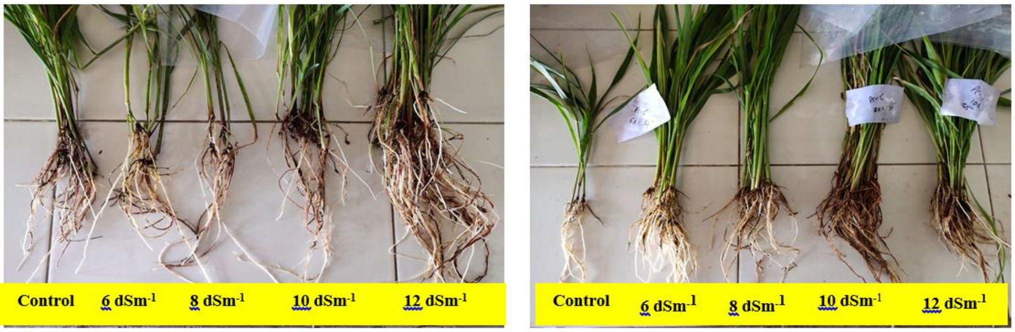

(b) **SSG 59-3** **PC-5**

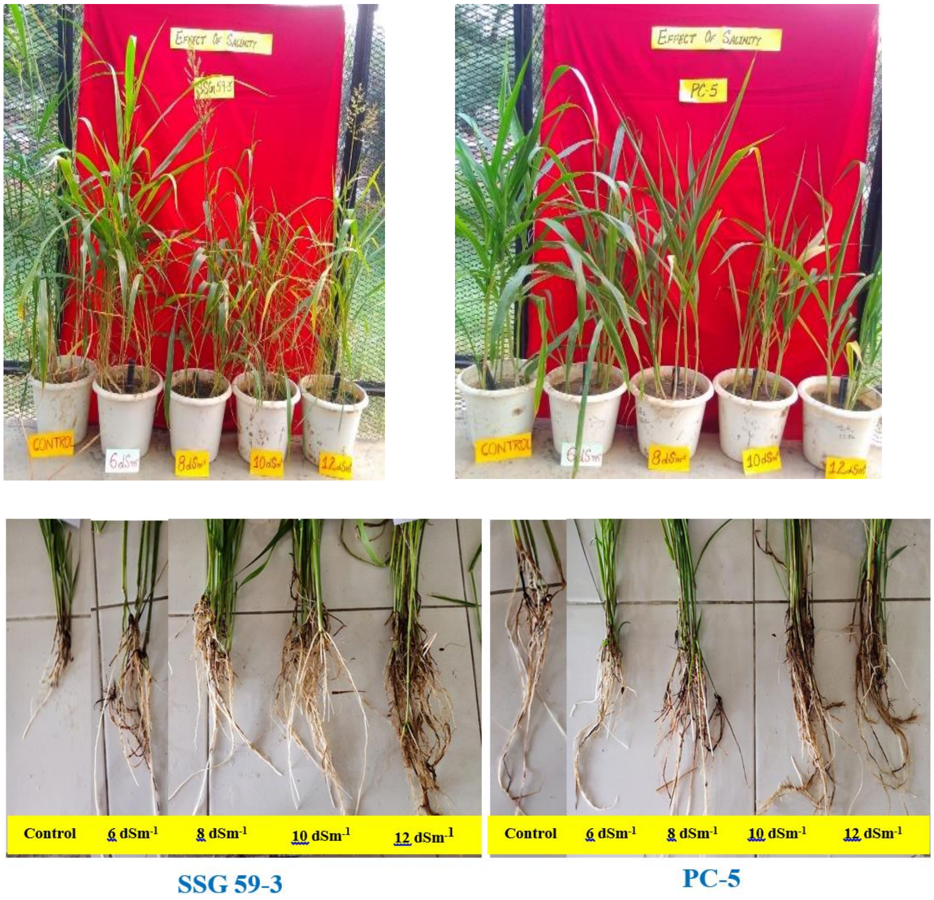

Figure S8. Effect of salt stress on the growth of sorghum genotypes (SSG 59-3 and PC-5) at (a) vegetative stage (35 DAS) and (b) physiological maturity (95 DAS).

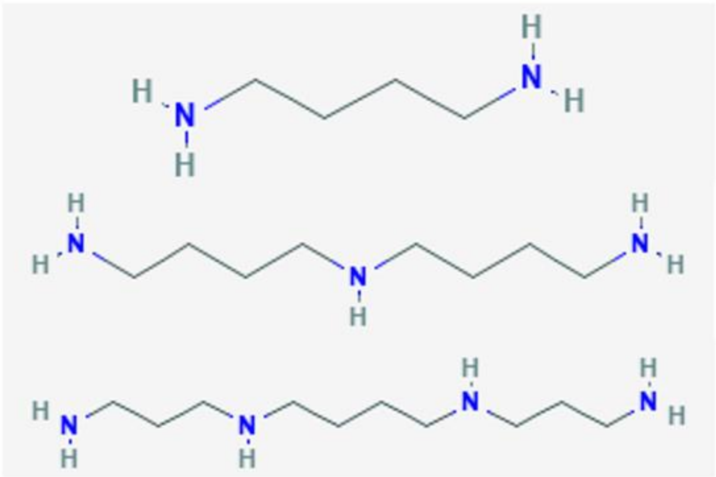

Put

Spd

Spm

Figure S9. Chemical structures of the polyamines discussed in this study.

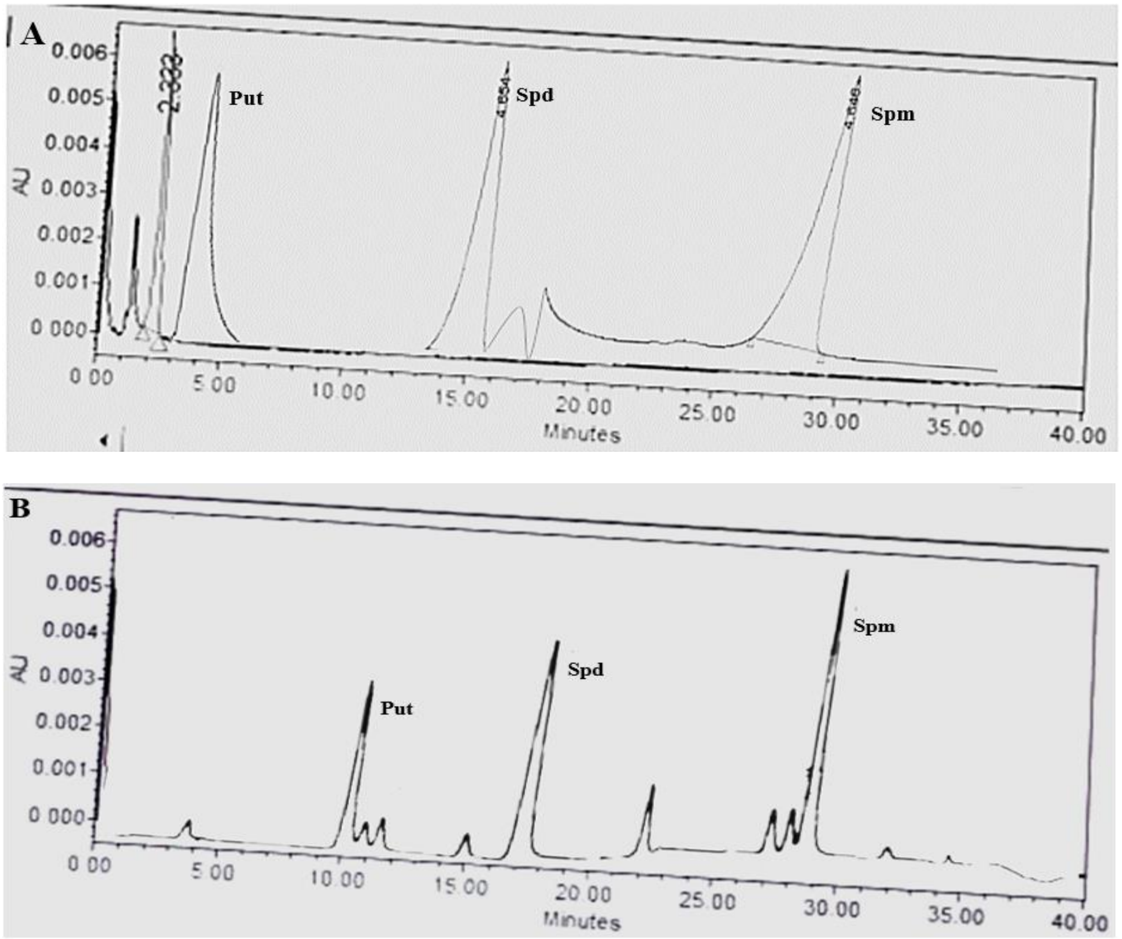

Figure S10. HPLC chromatogram and elusion profile of benzoylated polyamine (a) HPLC of benzoylated polyamine standards; Put, Spd, and Spm at 0.05mM concentration (b) Identification of polyamines in sorghum extracts with each of each Put, Spd, and Spm.

Supplementary Table S1. Physical characteristics of sorghum genotypes

Cultivars	Description	Source	Pedigree	Pericarp
**SSG 59-3**	Sweet Sudan Grass	CCS HAU Hisar, India	Non sweet sudan grass × JS-263	Red
**PC-5**	Pant Chari 5	GBPUA&T, Pantnagar, India	CS 3541 × IS 6953	Pearl White

Supplementary Table S2. Properties of soil in the pots under screen house experiment

Soil Texture	EC (%)	pH	OC (%)	N (mg/kg soil)	P (mg/kg soil)	K (mg/kg soil)
**Sandy-loam**	0.09	7.50	0.18	86.33	3.67	165.0

Supplementary Table S3. Composition of the saline solutions of different molarity

**Salts**	**The concentration of salts required (g/litre)**			
	**60mM**	**80 mM**	**100 mM**	**120 mM**
**NaCl**	1.10	2.34	2.92	3.51
**$CaCl_2.2H_2O$**	0.661	1.09	1.38	1.66
**$MgCl_2.6H_2O$**	0.549	0.610	0.762	0.915
**$MgSO_4.7H_2O$**	0.266	1.47	3.69	4.43
